# Case of Punctured Wound, with Tetanic Symptoms, Successfully Treated by Stimulants

**Published:** 1825-03

**Authors:** S. Nicholls


**Art. III.**—***Case of punctured Wound, with Tetanic Symptoms, successfully treated by Stimulants.*** By S. Nicholls, m.d.

Mr. Freeman, ætatis fifty one, an ingenious ornamental manufacturer, residing in Princes-street, Leicester-square, No. 42, on the 13th of October last, received accidentally a puncture from a small nail, in the anterior part of the last phalanx of the left thumb. The wound, being almost imperceptible, excited no observation until the following day, when it became painful and somewhat tumid. On the third day from the accident, after a restless night, he experienced acute pain and stiffness in the arm, with nausea and slight vertigo. On this day he consulted a medical practitioner, who advised the application of leeches to the thumb, a poultice, and brisk purgatives, together with rest, and a total abstinence from animal food. This plan was scrupulously pursued for four days; during which time, the general symptoms of rigor, inflammation along the course of the lymphatics of the arm, with intumescence, and acute pain extending from the thumb to the axilla, rapidly increased.

On the 20th, after a restless night, relieved only by short delirious slumbers, he awoke with a sense of rigidity of his jaw, and general stiffness of the affected limb. In the evening of that day I was consulted. His countenance exhibited extreme apprehension of the dangerous consequences likely to result from the accident; he had considerable tremor; and the slightest touch upon any part of the arm excited acute pain and involuntary shivering; slight pressure in the axilla occasioned a peculiar thrilling torture along the site of the radial and ulnar nerves, that extended to the thumb; his pulse was tense and wiry, at 100; and he complained of universal lassitude, thirst, and prostration of strength. Finding his power of articulation materially affected, and considering that the incipient stiffness of the jaw, and general tetanic symptoms, afforded positive proof of the rapid advance of genuine trismus, I resolved immediately to suspend the depletive plan, (which appeared to have been decidedly unsuccessful;) and, after endeavouring to relieve his mental anxiety, and establish his confidence in the measures I proposed to adopt, by assuring him of his speedy recovery, recommended his participating in a dinner of animal food, suited to his confined power of mastication. He drank about three parts in four of a bottle of white wine (sherry), and slept quietly for nearly an hour afterward; waking much refreshed.

At night he took for his supper, gruel, with an ounce of brandy; and a draught, containing forty drops of tincturæ opii. He passed a quieter night than usual, in a room warmed to the temperature of 62° Fahrenheit, and awakened in the morning with very slight rigidity of the jaw, and with much less pain and tension in the arm and hand: the thumb appeared, however, rather more swollen, with pulsatory throbbing, and the general indications of deep-seated suppuration. A large poultice of bread and milk was directed to be kept constantly applied, and frequently renewed; the cordial diet continued; and the following medicine prescribed:—℞. Ammon. carbon. gr. vj.; Ferri præcip. ℈j.; Tinct. cinch. comp.; Syr. papav. albi, āā ʒj.; Decocti cinchonæ, ℥x. M. fiat haustus ter in die sumendus.

Under this treatment (together with cheerful society in a family, the members of which endeavoured to subdue every apprehension of danger, by affecting to consider the disease comparatively trivial,) the tetanic symptoms gradually subsided; and, on the third day from its adoption, suppuration was evident extensively in the thumb, and particularly in the tendinous muscular structure forming the cushion of the upper phalanx, and beneath the tendons of the second. Deep and free incisions were made, transversely of the muscular fibres, wherever the suppuration had extended, with the double purpose of relieving the tension by the evacuation of the confined pus, and of dividing any twigs of the radial nerve, that might otherwise perpetuate the tetanic irritation. Although several small branches of the radial artery were divided in these operations, the hemorrhage was very slight, and unimportant.

On the sixth day from the commencement of the tonic plan, the arm presented a very satisfactory appearance: the red lines, marking the course of the lymphatics, were nearly obliterated, and the muscular rigidity and tension were almost imperceptible; the nervous sentience was materially obtunded; appetite and spirits good; pulse firm, at eighty; nights comfortable; and not the slightest affection of the jaw or face remaining.—The poultice was now discontinued; the cordial diet and tonic medicines were persevered in; and, excepting only that the thumb was tardy in healing, (a small part remaining uncicatrised for two months,) his restoration to health might be said to have been complete in six weeks from the accession of the accident.

I should not occupy your valuable pages beyond this concise statement, were it not to bear testimony to the correctness of Mr. Hutchinson's treatment of tic douloureux. I can speak with confidence to its propriety, from having adopted a similar plan of treatment, for upwards of twenty years past, in that and other cases of high nervous excitement. I have forborne transmitting a recital of cases, illustrative of the success that has resulted from the free exhibition of iron and other tonics in that terrible disease, lest it should appear that I claim any merit upon the score of priority in the practice. Mr. Hutchinson has the sole title to the thanks of the medical world, for announcing the fact. The *methodus medendi* is founded upon correct pathological induction, and, whenever it is judiciously adopted, will be successful.

*Hinton House, Twyford, Berks; Jan. 15, 1825.*

